# Predictors of women's sexual outcomes after implant‐based breast reconstruction

**DOI:** 10.1002/pon.5415

**Published:** 2020-06-05

**Authors:** Tim C. van de Grift, Marc A. M. Mureau, Vera N. Negenborn, Rieky E. G. Dikmans, Mark‐Bram Bouman, Margriet G. Mullender

**Affiliations:** ^1^ Department of Plastic, Reconstructive and Hand Surgery Amsterdam University Medical Center (VUmc) Amsterdam The Netherlands; ^2^ Department Medical Psychology and Sexology Amsterdam University Medical Center (VUmc) Amsterdam The Netherlands; ^3^ Amsterdam Public Health Institute Amsterdam The Netherlands; ^4^ Department of Plastic and Reconstructive Surgery, Erasmus MC Cancer Institute University Medical Center Rotterdam Rotterdam The Netherlands

**Keywords:** breast neoplasms, cancer, mammoplasty, oncology, orgasm, sexual behavior, sexual dysfunctions, social support

## Abstract

**Objective:**

Although breast reconstruction has become an important treatment modality following mastectomy, few studies assessed predictors of postoperative sexual outcomes after breast reconstruction. Therefore, we aimed to study three sexual outcomes following implant‐based breast reconstruction (IBBR), and associate multiple biopsychosocial factors with these outcomes.

**Methods:**

Data collection was part of a multicenter prospective study on IBBR. A predictive model was tested including medical, background and psychological predictors, partner relationship factors and physical sexual function. Data collection included clinical and questionnaire data (preoperatively and 1 year following reconstruction) using the BREAST‐Q Sexual well‐being scale (BQ5), and questions regarding sexual dysfunction and sexual satisfaction questions (Female Sexual Function Index).

**Results:**

The study sample consisted of 88 women who underwent mastectomy and IBBR. Mean postoperative BQ5 scores were lower than before surgery (M = 58 [SD = 18] vs 65 [SD = 20]; *P* = .01, Wilks' Lamdba = .88). Sexual dysfunctions were related strongest to orgasm inability and vaginal lubrication issues. The tested models predicted 37%‐46% of the sexual outcomes: sexual outcomes were mostly predicted by psychosocial well‐being, physical sexual function and partner support. Preoperative sexual and psychosocial well‐being were positively associated with postoperative sexual well‐being (*r* = 0.45 and *r* = 0.47).

**Conclusions:**

Although moderately positive sexual outcomes were reported after IBBR, some women reported issues with vaginal lubrication, breast sensation and orgasm. Sexual dysfunctions were predicted by vaginal lubrication and medical treatments, while sexual well‐being and satisfaction were more predicted by psychosocial well‐being and partner support. We advocate supportive care that includes partners and psychosocial functioning to optimize sexual outcomes after IBBR.

## 1 BACKGROUND

About one in eight women is diagnosed with breast cancer during their lives.^1^ With the substantial impact of surgical treatment on both physical health, as well as psychological coping, body image and femininity, the disease causes a significant burden for women’s psychosocial well‐being.^1^, ^2^ Because of improving survival rates, breast reconstructive surgery has gained importance within breast cancer treatments. Essentially, breast reconstruction aims to restore a feminine breast appearance by using implants or autologous tissue, improving the patient's quality of life.^2^

Sexual well‐being is an important part of quality of life^3^ and can be operationalized via sexual (dys)function, sexual satisfaction (appreciation in relation to the desired), and sexual well‐being (overall subjective experience). Although most previous studies focused on sexual dysfunctions, studying the different measures provides a differentiated view on sexual outcomes. A small body of literature focused on sexual outcomes after breast reconstruction specifically.^4^, ^5^, ^6^, ^7^, ^8^, ^9^, ^10^, ^11^, ^12^, ^13^, ^14^, ^15^ Sexuality is considered a biopsychosocial concept, and therefore is thought to be associated with both biological and psychosocial factors. Some studies emphasized sexuality and body satisfaction following breast reconstruction,^5^ as well as femininity and attractiveness.^6^ Other studies highlighted the positive role of nipple sparing^7^ or nipple reconstructing surgery^8^ on postoperative sexual outcomes. Immediate reconstruction was found preferable over delayed reconstruction regarding sexual outcomes.^9^ One study found no differences in body image and sexual relationship satisfaction between delayed autologous and implant‐based breast reconstruction (IBBR),^10^ while others found better sexual outcomes following autologous breast reconstruction.^11^

Two studies have reported an association between patient characteristics and sexual outcomes after breast reconstruction: older women reported higher sexual well‐being than younger women,^12^ whereas better preoperative quality of life and lower emotional distress predicted higher postoperative sexual well‐being.^13^ The partner also plays an important role in the process of breast reconstruction; couples greatly value partner involvement and joint surgical decision‐making.^14^ Partner intimacy after breast reconstruction was found to be related to couples’ adjustment and communication styles, and individual expectations.^15^

While some biological and some psychosocial factors have been associated separately to sexual outcomes, no comprehensive biopsychosocial analysis of predictors of sexual outcomes after breast reconstruction has been performed. Such knowledge would gain insight in how different aspects relate to each other, assisting clinicians in patient counseling and ultimately improve long‐term quality of life after IBBR.

### 1.1 Study objectives

**FIGURE 1:**
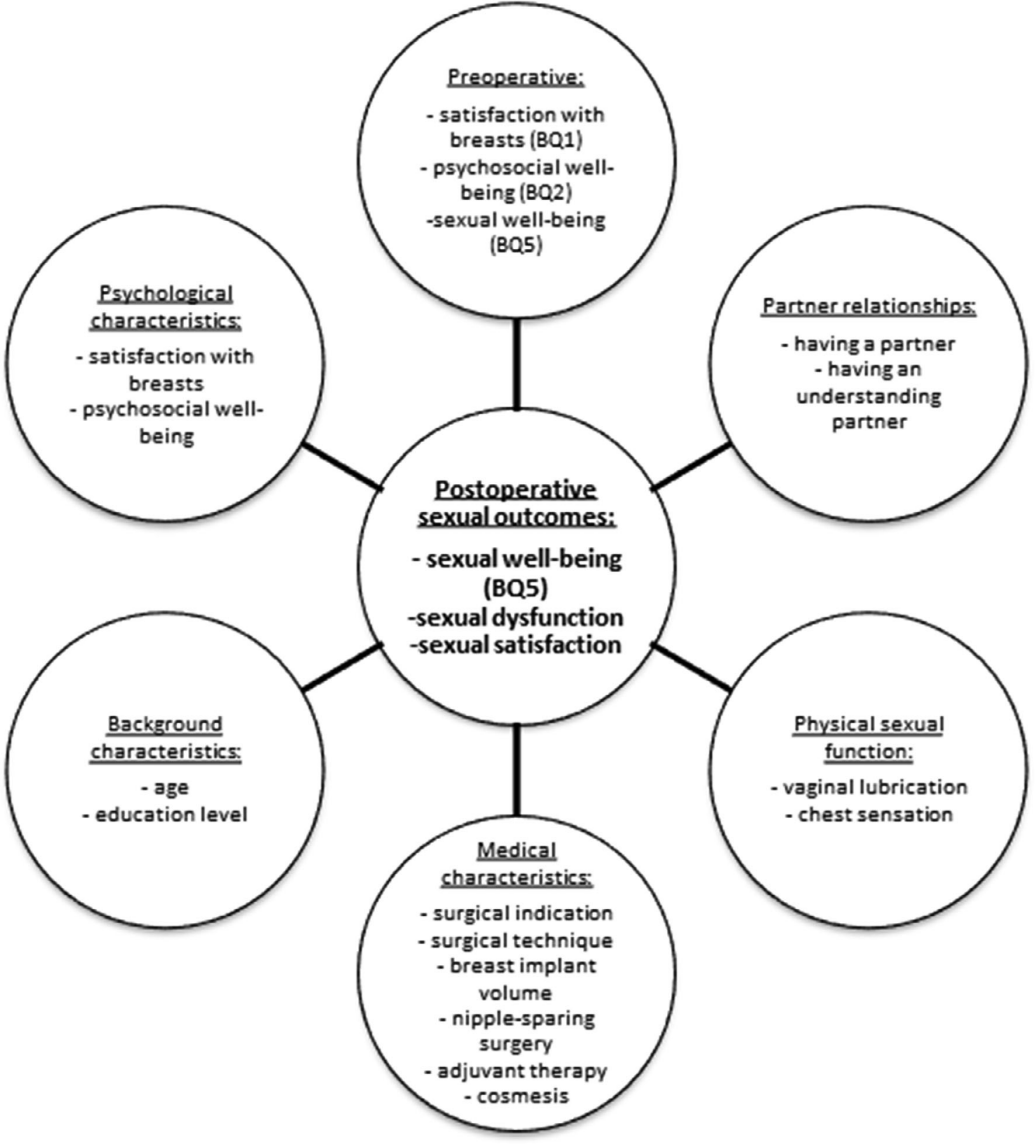
Conceptual model of predictors of sexual outcomes after breast reconstruction. Predictors include only postoperative variables, except for the preoperative BREAST‐Q (BQ) scores

The main objective of the current study was to assess sexual outcomes 1 year after IBBR by focusing on three measures; sexual well‐being (sexual quality of life), sexual dysfunction and sexual satisfaction. Following the results from the available literature, postoperative sexual outcomes were expected to be lower than before surgery. Furthermore, a biopsychosocial prediction model of postoperative sexual outcomes was designed based on the factors hypothesized in earlier studies and this model was tested in our sample (Figure 1).

## 2 METHODS

### 2.1 Conceptual framework

Based on the available literature and clinical expertise, a biopsychosocial framework of sexual outcome predictors after IBBR was developed (Figure 1). A detailed description of the factors is provided in the measures section.

### 2.2 Procedure

Data collection was part of the BRIOS study, a multicenter randomized controlled trial including women treated with an IBBR with either an implant and acellular dermal matrix, or a conventional two‐stage IBBR after tissue expansion.^16^ Women were recruited if they had a confirmed breast cancer or genetic predisposition, were at least 18 years old and intended to undergo a skin‐sparing mastectomy. Participants completed questionnaires preoperatively and 1 year after placement of the definite implant. Medical/surgical data were collected on case report forms, and standardized photographs were made before and after reconstruction. Ethical approval was obtained from all participating sites (all in the Netherlands; coordinating site: VU University Medical Center no. 2012/317) and was registered at the Netherlands Trial Register (no. NTR5446). More detailed descriptions of the study procedure have been published previously.^16^, ^17^

### 2.3 Participants

In the eight recruiting centers, 142 women consented to participate. Of this group, 20 women withdrew because of the surgical intervention they were randomized to, and one woman died because of a metastasized tumor, resulting in a final cohort of 121 participants. Participants were included for the present analyses if data were available on the main outcome measures, resulting in 88 participants (73%).

### 2.4 Main outcome measures

Postoperative sexual outcomes included three parameters:

**Sexual well‐being (BREAST‐Q)**: The BREAST‐Q is a widely‐used questionnaire to assess a range of quality of life domains after breast reconstruction surgery.^18^ The Sexual well‐being scale (BQ5) consists of six questions, surveying sexual attractiveness, confidence and comfort, which are transformed into a Q‐score ranging from 0 (worst) to 100 (best).

**Sexual dysfunction (FSFI)**: Three domains of subjective sexual dysfunction were assessed through a selection of questions from the Female Sexual Function Index (FSFI),^19^ in order to reduce the length of the entire survey: “During the past 4 weeks, how frequent did you feel aroused during sexual activity?,” “During the past 4 weeks, how often have you had an orgasm during sexual activity?” and “During the past 4 weeks, how often have you experienced an unpleasant/painful feeling during penetrative sex?” (all questions: 1 = (nearly) always, 2 = mostly, 3 = now and then, 4 = sometimes, 5 = (nearly) never). The three items showed acceptable internal consistency (Crohnbach's alpha = .63), and were averaged to one sexual dysfunction score.

**Sexual satisfaction (FSFI)**: Women were surveyed on their overall sexual satisfaction, using a single‐item question from the FSFI^19^: “During the past 4 weeks, how satisfied were you with your sex life in general?” (1 = very satisfied, 2 = somewhat satisfied, 3 = neither satisfied, nor dissatisfied, 4 = somewhat dissatisfied to 5 = very dissatisfied).

### 2.5 Other measures

Data collection used as predictors included (see Figure 1.):

**Questionnaire data**: This included participant’s age and highest education. Women stated whether they had a (sexual) partner, and if they experienced him/her as understanding (Maudsley Marital Questionnaire, from 0 = satisfied to 8 = largely missing^20^). Also, women completed the BREAST‐Q Satisfaction with Breasts (BQ1) and Psychosocial well‐being (BQ2; including body image, self‐esteem and emotional state) scales, resulting in 0 (worst) to 100 (best) Q‐scores.^18^ Lastly, women rated hypersensitivity of their breasts and vaginal lubrication during sexual activity (both: 1 = (nearly) never to 5 = (nearly) always).

**Clinical report form data**: Standardized data was collected on surgical indication (prophylaxis or malignancy), surgical approach (1‐ or 2‐stage), reoperations, nipple‐sparing or nipple reconstruction surgery, implant volume, and adjuvant chemotherapy and endocrine therapy.

The pseudonymized photos were evaluated independently by five plastic surgeons specialized in breast surgery. For each participant a mean panel score of the aesthetic outcome was collected, ranging from 1 (very poor) to 10 (very good).

### 2.6 Statistical analyses

Sample characteristics and the main outcomes were described as means (SD) or frequencies (percentages). Predictive model assessment, including postoperative variables, was performed in three steps:

1. Model construction and descriptive analysis of the background characteristics) and outcomes. Additional pre‐postoperative BREAST‐Q differences were calculated through repeated measures ANOVA.
2. Associations between all hypothesized predictors and the outcomes were explored via independent sample t‐tests to assess which factors to include into the final model (Table 3). A Bonferroni correction was applied to correct for multiple testing, and given the small sample size available for regression analysis only factors with statistical significance of *P* < .01 were put forward.
3. These factors were, as well as postoperative BQ1 and BQ2 values, entered into three linear regression models. Due to substantial missing values in the preoperative BREAST‐Q data, only simple pre‐postoperative correlations were calculated. Participants with (n = 50) and without (n = 38) preoperative data did not differ significantly in age, education level, relationship status and treatment characteristics.

All statistical analyses were performed in SPSS statistics 22.

## 3 RESULTS

### 3.1 Background characteristics

There were 88 participating women with a mean age of 45 years (range, 24 to 71 years), whose background characteristics are displayed in Table 1. Baseline mean breast satisfaction (BQ1) was 74 (SD = 18), psychosocial well‐being (BQ2) was 70 (SD = 16), and sexual well‐being (BQ5) was 65 (SD = 20).

**TABLE 1:** Sample characteristics (n = 88)

	n (%)
Mean age (in years, SD)	45 (12)
Highest education	
*Lower vocational*	11 (12.9)
*High school/higher vocational*	31 (36.5)
*College*	28 (32.9)
*University*	10 (11.8)
*No Diploma/Other*	5 (5.9)
Preoperative BREAST‐Q scale scores, mean (SD)^a^	
*Satisfaction with breasts (BQ1)*	74 (18)
*Psychosocial well‐being (BQ2)*	70 (16)
*Sexual well‐being (BQ5)*	65 (20)
Surgical indication	
*Prophylactic*	31 (35.2)
*Malignancy* ^b^	57 (64.8)
Surgical reconstruction technique	
*1‐Stage direct‐to‐implant with acellular dermal matrix*	48 (54.5)
*2‐Stage with tissue expander and breast implant*	40 (45.5)
Reoperation	
*No*	55 (62.5)
*Yes*	33 (37.5)
*Implant complication*	8
*Acellular dermal matrix removal*	6
*Aesthetic corrections* ^c^	11
*Other* ^d^	8
Adjuvant chemotherapy	25 (28.4)
Adjuvant endocrine therapy	25 (28.4)
Nipple‐sparing surgery	30 (34.1)
Nipple reconstruction	25 (28.4)
Implant volume in ml, mean (SD)	395 (108)
Panel score of postoperative aesthetic outcome, mean (SD, range)	6.3 (1.2, 1.6‐8.2)
Having a (Sexual) Partner	**75 (87.2)**
Having an understanding/warm partner^e^	**56 (75.7)**
Hyper sensation of the breasts^f^, mean (SD)	**1.82 (.90)**
Vaginal lubrication^g^, mean (SD)	**2.44 (1.24)**

*Note:* All variables include postoperative data, except the preoperative BREAST‐Q scores.

a BREAST‐Q scores range from 0 (worst outcome) to 100 (best outcome), data available for 51 participants.

b 46 ductal carcinoma, 8 DCIS, 1 LCIS, 1 Paget's Disease, 1 ductal carcinoma and DCIS.

c Necrosectomy, lipofilling, dogear correction.

d Removal of tissue expander, botox injection.

e Only calculated for women who reported having a (sexual) partner.

f Function scale ranges from 1 (never) to 5 (always).

g Dysfunction scales range from 1 ((nearly) always) to 5 ((nearly) never).

### 3.2 First study objective: Sexual outcomes after breast reconstruction

At follow‐up, the majority of women (87%) reported to have a (sexual) partner, most of whom experienced this partner to be understanding. On average, women reported moderately positive sexual well‐being (BQ5; M = 58, SD = 18), which was lower than before surgery (M = 65, SD = 20; *F*[1,49] = 6.98, *P* = .01, Wilks' Lamdba = 0.88). Judging from the mean scores, participants were moderately positive on their sex life in general (Table 2). Hypersensitivity of the breast(s) was experienced to a little extent, while issues with vaginal lubrication were more often reported.

**TABLE 2:** Sexual outcomes after breast reconstruction: sexual well‐being, (dys)functions and satisfaction

	Mean (SD)	Data available (n)
Sexual quality of life^a^		
Sexual well‐being (BQ5)	58 (18)	86
Sexual (dys)functions^b^		
Aroused during sexual activity	2.28 (1.21)	72 (+8)^d^
Orgasmic capacity during sexual activity	2.46 (1.33)	71 (+8)^d^
Absence of pain during penetrative sex	1.85 (1.25)	60 (+12)^d^
Mean sexual dysfunction score	2.25 (1.09)	73
Sexual satisfaction^c^		
Satisfaction with sex life in general	2.52 (1.20)	79

a BREAST‐Q (BQ) scores range from 0 (worst outcome) to 100 (best outcome).

b Dysfunction scales range from 1 ((nearly) always) to 5 ((nearly) never).

c Satisfaction scales range from 1 (very satisfied) to 5 (very dissatisfied).

d Participants with available data, but not sexually active or having a (sexual) partner or engaged in penetrative sex between brackets.

### 3.3 Second study objective: Assessment of biopsychosocial prediction model of sexual outcomes

Younger women reported worse sexual function outcomes compared to older participants (Table 3; *P* < .01, Cohen's *d* = 0.78). Women who had received a reoperation reported significantly lower BQ5 scores (*P* < .01, Cohen's *d* = 0.60). Women who had received prophylactic (Cohen's *d* = 0.59) or nipple sparing surgery (Cohen's *d* = 0.60), or who had not received chemotherapy (Cohen's *d* = 0.52) reported less sexual dysfunctioning (all *P* < .05). Women with a higher panel aesthetic score reported more sexual satisfaction (*P* < .05, Cohen's *d* = 0.57). Women who experienced infrequent/no vaginal lubrication, reported significantly unfavorable sexual function (*P* < .001, Cohen's *d* = 1.64) and satisfaction (*P* < .001, Cohen's *d* = 0.99). A similar trend was observed for an unfavorable BQ5 score (*P* < .05, Cohen's *d* = 0.49). Finally, no significant effect of having a (sexual) partner was found, while women with an understanding partner reported better BQ5 scores (*P* < .05, Cohen's *d* = 0.71) and sexual satisfaction (*P* < .01, Cohen's *d* = 0.91). No such difference was found for sexual dysfunction.

**TABLE 3:** Mean scores (SD) for predictors of postoperative sexual outcomes

Domain	Item	Sexual Well‐Being Score (BQ5)	Mean Sexual Dysfunction Score	Satisfaction with Sex Life in General
Background characteristics	Age *50 or Younger* *Above 50*	58 (17) 56 (19)	2.00 (1.00)** **2.80 (1.10)**	2.45 (1.10) 2.65 (1.39)
	Highest Education *Lower* *Intermediate* *Higher*	55 (20) 57 (17) 59 (19)	2.87 (0.84) 2.29 (1.24) 2.14 (0.99)	3.00 (1.23) 2.35 (1.25) 2.67 (1.16)
Medical characteristics	Surgical Indication *Prophylactic* *Malignancy*	58 (16) 57 (19)	1.85 (1.10)* 2.49 (1.02)	2.39 (1.31) 2.60 (1.13)
	Surgical Technique *1‐Stage with ADM* *2‐Stage with Implants*	58 (17) 57 (19)	2.24 (1.06) 2.26 (1.14)	2.66 (1.18) 2.34 (1.21)
	Reoperation *No* *Yes*	61 (18)** **51 (15)**	2.24 (1.06) 2.28 (1.14)	2.45 (1.26) 2.63 (1.10)
	Adjuvant CTx *No* *Yes*	59 (19) 54 (16)	2.10 (1.03)* 2.68 (1.16)	2.38 (1.18) 2.90 (1.18)
	Adjuvant Endx *No* *Yes*	59 (19) 54 (16)	2.15 (1.06) 2.56 (1.15)	2.40 (1.18) 2.82 (1.22)
	Nipple‐Sparing Surgery *No* *Yes*	55 (18) 62 (17)	2.46 (1.11)* 1.84 (0.93)	2.63 (1.22) 2.32 (1.16)
	Implant Volume *400 cc or Less* *Above 400 cc*	58 (19) 59 (15)	2.33 (1.08) 2.16 (1.12)	2.52 (1.21) 2.45 (1.15)
	Panel Score *6.0/10 and lower* *Above 6.0/10*	60 (16) 57 (19)	2.16 (0.88) 2.33 (1.20)	2.12 (1.11)* 2.78 (1.21)
Physical sexual function	Breast Hyper Sensation *(Almost) Never* *Regularly or Often*	59 (18) 50 (19)	2.23 (1.12) 2.36 (0.92)	2.42 (1.12) 3.00 (1.24)
	Vaginal Lubrication* *Sometimes or Never* *(Almost) Always*	53 (17)* 62 (18)	3.11 (1.11)*** **1.67 (0.53)**	3.03 (1.19)*** **1.98 (0.91)**
Partner relationship	(Sexual) Partner *No* *Yes*	54 (17) 58 (18)	1.60 (0.99) 2.32 (1.09)	2.80 (1.40) 2.50 (1.17)
	Understanding Partner *(Somewhat) Missing* *Mostly Satisfied*	50 (14)*** **61 (19)**	2.24 (1.30) 2.25 (1.00)	3.24 (1.15)*** **2.23 (1.08)**

*Note:* All predictors include postoperative data. ADM = Acellular Dermal Matrix, CTx = Chemotherapy, Endx = Endocrine Therapy. BREAST‐Q scores range from 0 (worst outcome) to 100 (best outcome); dysfunction score ranges from 1 ((nearly) always) to 5 ((nearly never); satisfaction scale ranges from 1 (very satisfied) to 5 (very dissatisfied). Factors included into the model are highlighted in bold; *P < 0.05, **P < 0.01, ***P < 0.001.

### 3.4 The final models

When entering the statistically significant factors into the prediction model, BQ5 was predicted for 46% by the first model (Table 4). BQ5 score was strongly associated with postoperative psychosocial well‐being (BQ1; *β* = 0.55, *P* < .001), while the other factors were of lesser importance. The second model predicted 46% of the mean sexual dysfunction score. Higher sexual dysfunction was predicted by a younger age (*β* = 0.55, *P* < .05) and more problems in vaginal lubrication (*β* = −0.61, *P* < .001). Satisfaction with sex life was predicted for 37% by the third model. Experiencing vaginal lubrication and having an understanding partner were equal predictors of sexual satisfaction (both *β* = −0.34, *P* < .01).

**TABLE 4:** Factors associated with postoperative sexual outcomes (*β*'s displayed)

	1.	2.	3.
	*Sexual Well‐Being Score (BQ5)*	*Mean Sexual Dysfunction Score*	*Satisfaction with Sex Life in General*
Model‐adjusted *R* ^2^	0.46	0.46	0.37
Model statistics	*F*(4,68) = 16.0, *P* < .001	*F*(4,66) = 15.7, *P* < .001	*P*(4,58) = 10.3, *P* < .001
**Cases included**	**73**	**71**	**63**
Age		**0.23** *	
Reoperation	−0.07		
Vaginal lubrication		**−0.61** ***	**−0.34** **
Understanding partner	0.09		**−0.34** **
Satisfaction with breasts	0.14	−0.10	−0.12
Psychosocial well‐being	**0.55** ***	0.03	−0.20

*Note:* All predictors include postoperative data. Significant factors in bold.

* *P* < .05.

** *P* < .01.

*** *P* < .001.

### 3.5 Pre‐ and postoperative associations

Higher postoperative sexual well‐being (BQ5) was significantly associated with more preoperative satisfaction with breasts (BQ1; *r*[51] = 0.40, *P* = .003), higher preoperative psychosocial well‐being (BQ2; *r*[51] = 0.45, *P* = .001) and higher preoperative sexual well‐being (BQ5; *r*[50] = 0.47, *P* = .001). No such associations were found for the sexual dysfunction and satisfaction outcomes.

## 4 DISCUSSION

The present study is the first to investigate the predictive effect of a range of biopsychosocial factors on sexual outcomes after mastectomy with IBBR. Key findings include a decrease in sexual well‐being after IBBR. The predictive factors showed different importance for each sexual outcome. Sexual dysfunctions were predicted by younger age and vaginal lubrication, whereas, sexual well‐being and satisfaction were less influenced by physical function or treatments. The sexual outcomes were most strongly predicted by (the quality of) partner relationships and psychosocial well‐being.

Preoperatively, study participants reported a relatively high mean sexual well‐being (BQ5), whereas this dropped by seven points postoperatively. These postoperative BQ5 scores are slightly higher than the published BQ5 reference values (1‐year postoperative *M* = 53 for IBBR).^2^ It was described before that postoperative BQ5 scores are generally lower than the other BQ domains and preoperative BQ5 scores.^3^ Possible explanations are that sexual rehabilitation may take a while and several other factors influence this process (eg, endocrine treatments, psychological adjustment). Possibly, the high threshold for seeking sexual counseling leads to poorer sexual outcomes as well.

We found that vaginal lubrication issues and patients’ age were the most important predictors of postoperative sexual dysfunction. Previous studies have highlighted the increased prevalence of lubrication and orgasm issues in women treated for breast cancer.^1^, ^4^ Vaginal lubrication issues may result from post‐treatment hormonal changes, as well as from lower arousability due to increased body awareness or psychological maladjustment. Insufficient vaginal lubrication can cause pain during penetration and difficulties reaching orgasm. Other factors influencing orgasmic ability include hormonal, psychological (eg, body image) and relationship factors.^15^ The positive association we observed between sexual outcomes and increasing age is in line with the findings of Santosa et al.^12^ Possibly, older women developed more mature/varied sexual repertoires with their partners, resulting in more arousal and orgasmic ability. Also, older couples may have more open communication skills, a factor that was emphasized before.^15^ Possible alternative explanations include a lower importance of physical ideals at an older age or higher sexual functioning amongst women who opt for reconstruction at a higher age.

Regarding the surgical predictors, we were only able to confirm the negative effect of having undergone reoperations on sexual dysfunction. Possibly, women who have had reoperation(s) experience more functional issues and higher psychological burden. Also, we found a small positive effect of nipple sparing surgery (in line with Wei et al^7^), possibly resulting from the preserved feminine appearance and sensation (although only limited nipple sensation preservation can be expected^21^). Finally, women with malignancy‐related surgery and women who received chemotherapy reported worse sexual functioning. It is known from literature that women who received chemotherapy and oncological surgery (in contrast to prophylactic surgery) are at risk for long‐term sexual function issues, due to vaginal dryness, body image issues and other mental health problems.^1^, ^2^

While vaginal dryness and medical treatments predicted sexual dysfunctions, contextual and psychosocial factors predicted sexual well‐being and satisfaction; none of the surgical factors showed statistical significance. In addition, vaginal lubrication also predicted sexual satisfaction. Ganz et al^1^ earlier observed this relationship between vaginal dryness and sexual outcomes after breast cancer treatment. It is important for clinicians to pay attention to such physical sexual dysfunctions and offer proper treatments (eg, sexual counseling or lubricants) to whoever needs it.

Having an understanding partner was important for both postoperative sexual well‐being and satisfaction. Other studies observed that partner involvement was important in the preoperative decision‐making in women choosing breast reconstruction.^14^ Also, partner‐involvement supports dyadic adjustment^1^ and couple (sexual) communication.^15^ Therefore, we advocate the involvement of partners during surgical decision‐making and postoperative follow‐up.^22^ Investing in couple adjustment and communication can support women in developing better postoperative sexual outcomes.

Postoperative sexual well‐being was strongly associated with preoperative sexual and psychosocial well‐being, which corroborates the findings of previous studies.^1^, ^13^ Women experiencing low preoperative sexual well‐being may very well report persisting issues postoperatively. Psychosocial issues can induce additional sexual problems through mood disorders (lowered sexual drive) and body image issues (lowered sexual engagement), whereas socio‐economic stress may impact both psychosocial and sexual well‐being.

### 4.1 Study limitations

The study was limited by the sample size, which may not have allowed to detect smaller effects and to test all possible predictors in the regression analysis. Also, not all study participants filled out preoperative measures, possibly resulting in selection bias (eg, women experiencing problems may have been more willing to participate, while the opposite cannot be ruled out either). For some measures, self‐constructed questions were used instead of standardized instruments, which may have reduced sensitivity. Additionally, questionnaire studies are prone to cognitive biases; for example, in our study women may have filled out measures similarly, regardless of the measured construct. Another study limitation is that only implant‐based groups were compared, and no comparison with other surgical techniques (eg, autologous reconstruction) could be made. Clinically, it is important to notice that access to sexual counseling greatly varies per geographical location, and that this study was conducted in a country with access to partially funded sexual counseling.

Strengths of the present study include the prospective design and comprehensive approach, allowing to assess how the different predictors compare. Also, the study provides clinically‐relevant topics to assist professionals in this field.

### 4.2 Clinical implications

Breast reconstruction influences women's sexuality. Subgroups at risk for developing poorer sexual outcomes include younger women, women with fewer partner support, women with preexistent poor psychosocial well‐being, and women with postoperative vaginal dryness. Sexual function issues are known (long‐term) effects of cancer treatments that may not dissolve naturally without clinical counseling. Our findings underline the importance of assessing sexual outcomes after IBBR and we advocate a holistic approach including mental health and partners into treatments.

## 5 CONCLUSIONS

Following mastectomy and IBBR, women reported moderately positive sexual outcomes. At the same time, some experienced problems with physical function and orgasm. While sexual dysfunctions were predicted by women’'s younger age and vaginal lubrication, sexual well‐being and satisfaction were predicted by partner understanding and general psychosocial well‐being. None of the outcomes were predicted by surgical characteristics primarily. An integrated approach after breast reconstruction is advocated to support postoperative sexual outcomes.

## Data Availability

The data that support the findings of this study are available from the corresponding author upon reasonable request.
